# Risk of Incident Cardiovascular Disease and Cardiovascular Risk Factors in First and Second-Generation Indians: The Singapore Indian Eye Study

**DOI:** 10.1038/s41598-018-32833-0

**Published:** 2018-10-04

**Authors:** Preeti Gupta, Alfred Tau Liang Gan, Ryan Eyn Kidd Man, Eva K. Fenwick, Yih-Chung Tham, Charumathi Sabanayagam, Tien Yin Wong, Ching-Yu Cheng, Ecosse L. Lamoureux

**Affiliations:** 1 Singapore Eye Research Institute, Singapore, Singapore; 2 Duke-NUS Medical School, Singapore, Singapore; 3 Singapore National Eye Centre, Singapore, Singapore; 4 National University of Singapore, Department of Ophthalmology, Singapore, Singapore

## Abstract

Population-based data investigating generational differences in the risk of incident cardiovascular disease (CVD) and its risk determinants are rare. We examined the 6-year incidence of CVD and its risk factors in first- and second-generation ethnic Indians living in Singapore. 1749 participants (mean age [SD]: 55.5 [8.8] years; 47.5% male) from a population-based, longitudinal study of Indian adults were included for incident CVD outcome. Incident CVD was defined as self-reported myocardial infarction, angina pectoris or stroke which developed between baseline and follow-up. CVD-related risk factors included incident diabetes, hypertension, hyperlipidemia, obesity and chronic kidney disease (CKD). For incident CVD outcome, of the 1749 participants, 406 (23.2%) and 1343 (76.8%) were first and second-generation Indians, respectively. Of these, 73 (4.1%) reported incident CVD. In multivariable models, second-generation individuals had increased risk of developing CVD (RR = 2.04; 95% CI 1.04, 3.99; p = 0.038), hyperlipidemia (RR = 1.27; 95% CI 1.06, 1.53; p = 0.011), and CKD (RR = 1.92; 95% CI 1.22, 3.04; p = 0.005), compared to first-generation Indians. Second-generation Indians have increased risk of developing CVD and its associated risk factors such as hyperlipidemia and CKD compared to first-generation immigrants, independent of traditional CVD risk factors. More stratified and tailored CVD prevention strategies on second and subsequent generations of Indian immigrants in Singapore are warranted.

## Introduction

Previous research has shown that cardiovascular disease (CVD), which accounts for approximately 31% of global mortality rates^1^, is affected by environmental, lifestyle and risk factors such as obesity and physical inactivity^2–4^. It has been hypothesized that acculturation (the process of adaptation and exchange of behavior patterns to the principal culture in the new country) accelerates these factors^5–8^, corroborated by research showing that individuals migrating to a new country have suboptimal lifestyle profiles such as sedentariness^9^, and a high consumption of energy dense food^10–12^, compared to those in the home country, leading to dysfunctional metabolic states including obesity, hypertension, diabetes, and hyperlipidemia, culminating in an increased risk of CVD^13–15^.

While cross-sectional studies have reported higher CVD risk and poor cardiovascular profiles in more acculturated second-generation (born in host country) individuals compared to less acculturated foreign-born immigrants^16–23^, the observed generational variations do not provide an estimate of the “true” generational effect on health, as they may be confounded by secular trends in the health of immigrants. Currently, longitudinal data on the impact of generational changes on the incidence of CVD and its risk factors in immigrant populations, particularly from Asian cultures, are rare. It is possible that the impact of acculturation on the incidence of CVD and its risk factors differs in Asian compared to Western populations due to differences in healthcare systems and lifestyle, cultural, religious and environmental habits, as well as differences in the way that people perceive and cope with illness and disease^24,25^. Addressing this knowledge gap can inform the temporal relationship between generational status and the development of CVD and its risk determinants, and health care agencies likely to target second-generation individuals in their prevention towards development of CVD and its risk determinants.

Such information is particularly important for Asian Indians who is one of the fastest growing immigrant groups across Asia and the world^26^. Singapore is a major migration destination, with Indian immigrants accounting for ~10% of Singapore’s total population^27,28^. As such, the Singapore population is ideal to study generational differences in chronic diseases including CVD.

In this study, we evaluated the 6-year incidence of CVD and its risk factors between first- and second-generation Indians in Singapore. We hypothesize that second-generation Indians will have a higher incidence of CVD and its risk predictors than first-generation immigrants. Findings from this study may potentially inform how nativity is related to change in cardiovascular health over time, and advise generation specific interventions to reduced future CVD risk.

## Methods

### Study Population and Design

The Singapore Indian Eye Study (SINDI-1 and -2) is a population-based cohort study of Indian adults (aged 40–80 years) living in Singapore, with baseline and follow-up assessments conducted between 2007 and 2015^29,30^. Briefly, from a list of 12,000 individuals living in south-western Singapore, an age-stratified random sampling method selected 6350 names, of which 4168 were eligible. A total of 3400 (75.6% response rate) participated in SINDI-1. After 6-years, 486 (14.2%) of the initial subjects were ineligible to participate in SINDI-2 due to cognitive and/or severe mobility impairments, migration to other countries, or death during the intervening 6-years. Of the remaining 2914 eligible participants, 2200 (75.5% response rate) participated in SINDI-2.

The study was conducted at the research clinic of the Singapore Eye Research Institute (SERI). The protocol included a comprehensive, standardized examination as well as a questionnaire administered by interviewers trained to collect clinical and socio-demographic data, as described previously^29,30^. All protocols followed the principles of the Declaration of Helsinki and received ethics approval by the SingHealth Institutional Review Board. Written informed consent from participants was obtained prior to participation in the study. Data can be made available upon request from the corresponding author.

### Definition of Immigrant Status

Each participant’s ethnicity was confirmed by using data from the Singapore national identity card. Each was categorized as “first-generation Indian” if he/she was born in India or Indian subcontinent, irrespective of the country of birth of their parents; or “second-generation Indian” if born in Singapore or Malaysia, irrespective of the country of birth of their parents.

### Assessment of CVD

CVD information was collected using an interview-based, standardized questionnaire at the baseline and follow-up examinations. CVD was defined as a participant’s self-reported history of myocardial infarction, angina pectoris, or stroke, similar to previous epidemiologic studies conducted by our group^31^. Incidence of CVD was defined as no CVD at baseline examination but present at follow-up. We performed a reliability assessment of our self-reported CVD outcome and found that 75.5% of those who reported a history of CVD at baseline confirmed this information in the follow-up examination as reported elsewhere^31^.

### Assessment of CVD Risk Factors

Socio-demographic characteristics (educational level, income level, occupation), lifestyle factors (e.g. smoking, alcohol consumption), self-reported family and medical history (e.g. diabetes, hypertension, stroke, and CVD), and current medications were obtained by trained interviewers through interviews.

Clinical covariates were obtained through a standardized clinical examination. Systolic and diastolic blood pressure (SBP and DBP) measurements were taken 2 times (a third if needed) using a digital BP monitor (Dinamap Pro Series DP110X-RW; GE Medical Systems Information Technologies, Inc). The average value of the two closest values for each parameter was utilized in analyses. Height and weight were measured using a wall-mounted adjustable measuring scale and a calibrated scientific weight scale, respectively. Body mass index (BMI) was calculated as weight (kg) divided by height in meters squared (kg/m^2^), and obesity was defined as BMI ≥ 30 kg/m^2^.

Non-fasting venous blood samples were collected to assess levels of glucose, haemoglobin A_1c_ (HbA_1c_), serum creatinine, serum total cholesterol, high-density lipoprotein cholesterol, low-density lipoprotein cholesterol, and triglycerides. All samples were analyzed at the Singapore General Hospital Hematology Laboratory.

Diabetes was defined as a random glucose ≥ 11.1 mmol/L, HbA1c ≥ 6.5% (≥48 mmol/moL), self-reported use of diabetic medication, or history of physician-diagnosed diabetes^32^; hypertension as SBP ≥ 140 mm Hg, DBP ≥ 90 mm Hg, physician’s diagnosis, or use of anti-hypertensive medication^33^; hyperlipidemia as total cholesterol ≥6.2 mmol/L or use of lipid lowering drugs and chronic kidney disease (CKD) as having an estimated glomerular filtration rate of <60 mL/min/1.73 m^2^ ^34,35^. For the incident CVD risk factor analysis, incidence of diabetes, hypertension, hyperlipidemia, obesity and CKD was defined as absent at baseline examination, but present at follow-up.

### Statistical Analyses

All analyses were performed using Stata version 14.2 (Statacorp, Lake Station, TX, USA). Baseline characteristics of participants by immigration status (first versus second-generation), and participants who returned and did not return for follow-up were compared using the χ^2^ statistic for proportions for categorical variables, and a t test for continuous variables. The Mann-Whitney U test was performed if the distribution of a continuous variable was highly skewed. (Tables 1 and 2, respectively**)**. Modified Poisson regression was used to determine whether immigrant status was independently associated with development of CVD and its risk factors (Tables 3 and 4, respectively**)**. Models were adjusted for participant characteristics that were either significantly different between generations at baseline or have been previously found to be associated with CVD and its risk factors. For each CVD condition in question, we also mutually adjusted for the other CVD conditions to check for an independent effect of that condition. Key covariates included age, gender, obesity, socioeconomic status (SES; including education [≤6 years/>6 years], income [<SG$2000/≥ SG$2000] and housing [≤3–4 room/> 3–4 room]), smoking status, alcohol and medication use (including anti diabetic, hypertensive or lipid lowering), and presence of comorbid conditions such as diabetes, hypertension, hyperlipidemia and CKD. Relative risks (RR) were reported with 95 percent confidence intervals (CI), and a two-tailed p-value of <0.05 was judged statistically significant.

**Table 1:** Comparison of baseline characteristics of participants by generation status in SINDI (N = 1749^#^).

Variable	Mean (SD) or Number (%)		
	1^st^ Generation (n = 406)	2^nd^ Generation (n = 1343)	P value*
Age, years	58.1 (10.2)	54.8 (8.2)	**<0.001**
Length of stay in Singapore, years	35.3 (17.0)	52.1 (9.8)	**<0.001**
Gender, Male	212 (52.2)	621 (46.2)	**0.035**
Obesity (BMI ≥ 30 kg/m^2^)	52 (12.8)	241 (17.9)	**0.015**
Low socioeconomic status	161 (39.7)	646 (48.1)	**0.003**
Current smoker	30 (7.4)	189 (14.1)	**<0.001**
Alcohol drinker	43 (10.6)	173 (12.9)	0.219
Diabetes	141 (34.7)	454 (33.8)	0.731
Hypertension	223 (54.9)	687 (51.2)	0.182
Hyperlipidemia	157 (38.7)	583 (43.4)	0.090
Chronic kidney disease	22 (5.4)	61 (4.5)	0.467
Anti-diabetic medication use	104 (25.6)	292 (21.7)	0.102
Anti-hypertensive medication use	137 (33.7)	389 (29.0)	0.066
Anti-cholesterol medication use	111 (27.3)	322 (24.0)	0.169

*Mann-Whitney U test or chi-squared test.

^#^1749 individuals had no CVD at baseline, had complete data for all baseline characteristics including generation status and returned for follow-up.

Low socioeconomic status was defined as primary or lower education, and individual monthly income < SGD2000.

Bolded values indicate statistically significant results.

**Table 2:** Comparison of baseline characteristics of participants by follow-up status.

Variable	Mean (SD) or Number (%)		
	Lost to follow-up (n = 854)	Returned for follow-up (n = 1749)	P value*
Age, years	58.3 (10.8)	55.6 (8.8)	**<0.001**
Length of stay in Singapore, years	49.1 (15.1)	48.2 (13.8)	0.099
Gender, Male	412 (48.2)	833 (47.6)	0.768
Obesity (BMI ≥ 30 kg/m^2^)	165 (19.3)	293 (16.8)	0.106
Low socioeconomic status	469 (54.9)	807 (46.1)	**<0.001**
Current smoker	155 (18.1)	219 (12.5)	**<0.001**
Alcohol drinker	112 (13.1)	216 (12.3)	0.581
Diabetes	342 (40.0)	595 (34.0)	**0.003**
Hypertension	529 (61.9)	910 (52.0)	**<0.001**
Hyperlipidemia	370 (43.3)	740 (42.3)	0.623
Chronic kidney disease	85 (10.0)	83 (4.7)	**<0.001**
Anti-diabetic medication use	212 (24.8)	396 (22.6)	0.217
Anti-hypertensive medication use	291 (34.1)	526 (30.1)	**0.039**
Anti-cholesterol medication use	212 (24.8)	433 (24.8)	0.970

^*^Mann-Whitney U test or chi-squared test.

Low socioeconomic status was defined as primary or lower education, and individual monthly income < SGD2000.

Bolded values indicate statistically significant results.

**Table 3:** Association between generation and incidence of CVD and its sub-types.

	Number^†^	Incident cases^‡^ (%)	Age-gender adjusted		Multivariable-adjusted*	
			Relative risk (95% CI)	P value	Relative risk (95% CI)	P value
**CVD**						
1^st^ Generation	406	10 (2.5)	Reference = 1		Reference = 1	
2^nd^ Generation	1343	63 (4.7)	2.34 (1.20 to 4.53)	**0.012**	2.04 (1.04 to 3.99)	**0.038**
Myocardial infarction						
1^st^ Generation	455	5 (1.1)	Reference = 1		Reference = 1	
2^nd^ Generation	1486	18 (1.2)	2.89 (1.11 to 7.52)	**0.029**	2.26 (0.87 to 5.89)	0.095
Stroke						
1^st^ Generation	420	5 (1.2)	Reference = 1		Reference = 1	
2^nd^ Generation	1407	40 (2.8)	2.02 (0.76 to 5.40)	0.161	2.01 (0.76 to 5.36)	0.161
Angina						
1^st^ Generation	443	4 (0.9)	Reference = 1		Reference = 1	
2^nd^ Generation	1442	21 (1.5)	1.73 (0.58 to 4.53)	0.325	1.73 (0.56 to 5.38)	0.345

^*^Adjusted for age, gender, obesity, low SES (primary or lower education, and individual monthly income < SGD2000), smoking, alcohol, presence of comorbid conditions such as diabetes, hypertension, hyperlipidemia and CKD, use of anti-diabetic, anti-hypertensive and anti-cholesterol medications.

Bolded values indicate statistically significant results.

^†^The number of individuals at risk of CVD is not the sum of the number of individuals without each of the constituent conditions (myocardial infarction, stroke and angina) at baseline. For example an individual without myocardial infarction at baseline (at risk of developing incident myocardial infarction) might already have angina and/or stroke and wouldn’t be included in the denominator for CVD.

^‡^Number of incident CVD cases is also not the sum of the incidence cases of each constituent condition as individuals could develop more than one condition.

CVD = cardio vascular disease; CKD = chronic kidney disease; SES = socioeconomic status.

**Table 4:** Association between generation and incidence of CVD related conditions.

	Number^#^	Incident Cases (%)	Age-gender adjusted		Multivariable-adjusted*	
			Relative risk (95% CI)	P value	Relative risk (95% CI)	P value
Diabetes						
1^st^ Generation	290	35 (12.1)	Reference = 1		Reference = 1	
2^nd^ Generation	966	137 (14.2)	1.18 (0.83 to 1.68)	0.345	1.16 (0.81 to 1.66)	0.407
Hypertension						
1^st^ Generation	186	62 (33.3)	Reference = 1		Reference = 1	
2^nd^ Generation	681	256 (37.6)	1.13 (0.90 to 1.41)	0.293	1.07 (0.85 to 1.34)	0.573
Hyperlipidemia						
1^st^ Generation	242	87 (36.0)	Reference = 1		Reference = 1	
2^nd^ Generation	765	351 (45.9)	1.33 (1.11 to 1.60)	0.002	1.27 (1.06 to 1.53)	0.011
CKD						
1^st^ Generation	403	22 (5.5)	Reference = 1		Reference = 1	
2^nd^ Generation	1378	83 (6.0)	1.93 (1.22 to 3.03)	0.005	1.92 (1.22 to 3.04)	0.005
Obesity						
1^st^ Generation	403	18 (4.5)	Reference = 1		Reference = 1	
2^nd^ Generation	1240	76 (6.1)	1.19 (0.72 to 1.97)	0.487	1.26 (0.76 to 2.11)	0.374

^*^Adjusted for age, gender, obesity, low SES (primary or lower education, and individual monthly income < SGD2000), smoking, alcohol, use of anti-diabetic, anti-hypertensive and anti-cholesterol medications, CVD and mutually for the other CVD related conditions at baseline.

^#^Number at risk varied depending on outcome (e.g. only 867 participants did not have hypertension at baseline and were at risk of incident hypertension).

Bolded values indicate statistically significant results.

CVD = cardio vascular disease; CKD = chronic kidney disease; SES = socioeconomic status.

## Results

Of the 3400 individuals who participated in SINDI-1, 3022 (88.8%) had complete data for baseline characteristics including information on immigrant status as per our definitions. Of these, 1981 (65.5%) returned for follow-up examination. Of these, 232 (11.7%) had CVD at baseline, leaving 1749 (88.3%) individuals (with no CVD at baseline, information on immigrant status and other relevant covariates at baseline and CVD data at follow-up) for the final analysis of incident CVD outcome. Of these, 406 (23.2%) and 1343 (76.8%) were first- and second-generation Indians, respectively. For the analysis of the association between immigrant status and incident CVD risk factors, study populations for incident diabetes, hypertension, hyperlipidemia, obesity and CKD were 1256, 867, 1007, 1643 and 1781, respectively.

Table 1 shows the differences in baseline characteristics of participants by immigration status (first versus second-generation). Compared to first (n = 406, 23.2%), second-generation individuals (n = 1343, 76.8%) were younger, more likely to be female and to be obese, have low SES (primary or lower education, and individual monthly income <SGD2000), were smokers and have a longer duration of stay in Singapore.

Table 2 shows the differences in baseline characteristics of participants who returned for follow-up versus those who did not. Compared to those who returned for follow-up (n = 1749, 67.2%), those lost to follow-up (n = 854, 32.8%) were older, more likely to be smokers, have low SES, have other comorbid conditions such as diabetes, hypertension, CKD and were more likely to be on antihypertensive medications.

**Figure 1:**
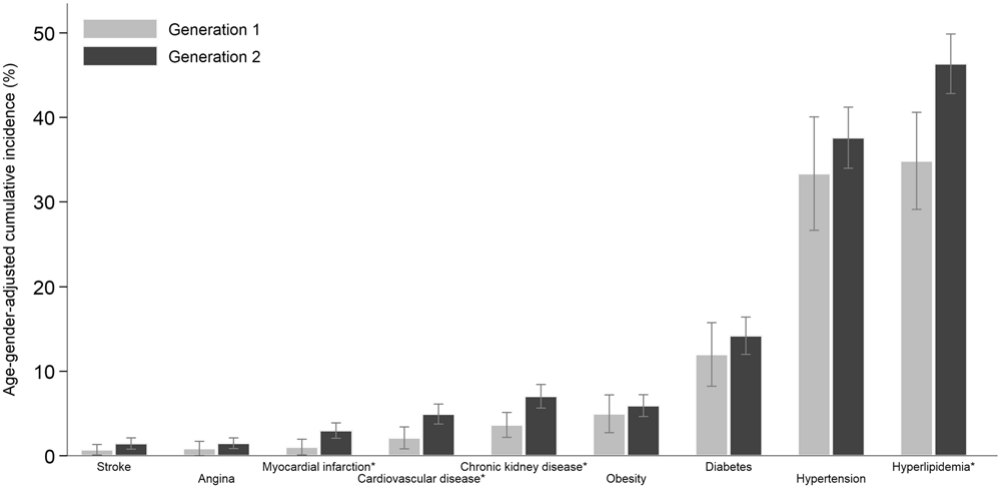
6-year cumulative incidence (age and gender adjusted) of CVD and related conditions in first- and second-generation Indians in Singapore. Asterisk indicates statistical significance between generations (p < 0.05). Error bars represent 95% confidence intervals.

Of the 1749 participants included in our final analyses for incident CVD (mean age (SD) 55.5 (8.8) years); 833 (47.5%) male), 73 (4.1%) developed CVD during the 6-year follow-up period. Figure 1 shows the 6-year cumulative incidence (age and gender adjusted) of CVD and its risk factors in first- and second-generation Indians in Singapore. Compared with first-generation, second-generation Indians had significantly higher incidence of myocardial infarction, CVD, CKD and hyperlipidemia.

**Figure 2:**
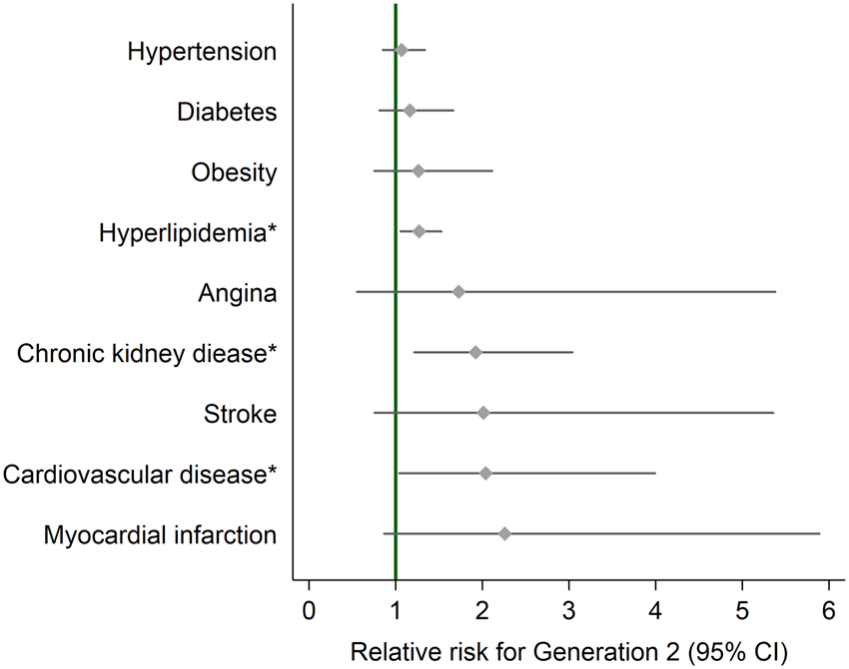
Relative risk of incident CVD and related conditions in second versus first-generation Indian individuals in Singapore. Asterisk indicates statistical significance (p < 0.05). Error bars represent 95% confidence intervals. Relative risk estimates have adjusted for age, gender, obesity, socioeconomic status, smoking, alcohol use, anti-diabetic, anti-hypertensive, and anti-cholesterol medications use, and mutually for the other CVD related conditions at baseline.

Table 3 shows the multivariable adjusted models for the impact of generation on the incidence of CVD and its sub-types. In model 1 (adjusted for age and gender), compared to first-generation immigrants, second-generation individuals had more than a two-fold increased risk of developing CVD (RR = 2.34; 95% CI 1.20, 4.53; p = 0.012). These findings persisted in a fully adjusted model accounting for age, gender, obesity, low SES, smoking status, alcohol use, presence of comorbid conditions such as diabetes, hypertension and hyperlipidemia, and use of anti-diabetic, anti-hypertensive and anti-cholesterol medications (RR = 2.04; 95% CI 1.04, 3.99; p = 0.038; model 2 in Table 3 and Fig. 2). Upon further sub-analysis of CVD subtypes (myocardial infarction, stroke or angina), no significant association between generation and CVD subtypes was seen (all p > 0.05; model 2 in Table 3, Fig. 2).

Table 4 shows the multivariable models for the impact of generation on the incidence of CVD risk factors including, diabetes, hypertension, hyperlipidemia, obesity, and CKD. In model 1 (adjusted for age and gender), compared to first-generation immigrants, second-generation individuals had 1.33 and 1.93 times increased risk of developing hyperlipidemia (RR = 1.33; 95% CI 1.11, 1.60; p = 0.002) and CKD (RR = 1.93; 95% CI 1.22, 3.03; p = 0.005), respectively. These findings persisted in a fully adjusted model accounting for age, gender, SES, smoking, alcohol, medication use, CVD, and mutually for other CVD related conditions at baseline including diabetes, hypertension, hyperlipidemia, obesity and CKD, for both hyperlipidemia (RR = 1.27; 95% CI 1.06, 1.53; p = 0.011) and CKD (RR = 1.92; 95% CI 1.22, 3.04; p = 0.005; model 2 in Table 4 and Fig. 2).

Supplementary Table S1 shows the multivariable models for the association of length of stay in Singapore with the incidence of CVD and its risk factors including, diabetes, hypertension, hyperlipidemia, obesity, and CKD. In a fully adjusted model accounting for age, gender, obesity, low SES, smoking, alcohol, use of anti-diabetic, anti-hypertensive, and anti-cholesterol medications, and mutually for the other CVD related conditions at baseline, a longer length of stay in Singapore was independently associated with higher incidence of hyperlipidemia (RR = 1.12; 95% CI 1.05, 1.20; p = 0.001) and CKD (RR = 1.26; 95% CI 1.07, 1.48; p = 0.005).

## Discussion

In this prospective, population-based study of ethnic Indian adults living in Singapore, a significant generational difference in incidence of CVD and its risk factors was found. We demonstrated that second-generation Indian individuals had increased risk of incident CVD, hyperlipidemia and CKD compared to first-generation immigrants, independent of conventional cardiovascular risk factors. Our findings highlight the need of targeted CVD and its risk factors preventative strategies in high-risk second-generation Indian individuals, potentially translatable not only to Singaporean Indians but also to other migrant Indians living outside India.

To the best of our knowledge, this is the first longitudinal study to examine the impact of generation on incidence of CVD and its major risk factors in Asians, making it challenging to compare our findings. Nevertheless, our results are consistent with longitudinal studies reporting lower incidence of CVD among foreign-born than US-born individuals^36,37^. Our findings confirm the growing body of evidence from previous cross-sectional studies that demonstrated higher prevalence of CVD, its conventional risk factors, and potentially modifiable CVD risk factors such as diet, physical inactivity and psychological stress in more acculturated second-generation (born in host country) individuals than less acculturated first-generation (born in home country) immigrants^20,28,38–40^.

Consistent with previous work, our findings also suggest that longer length of stay in host country may be associated with higher rates of CVD (but not CVD subtypes likely due to inadequate statistical power) and its risk factors. Studies have demonstrated that individuals residing for a long time in host country have higher physiological and psychological stress compared to those with fewer years of residence^41^. This finding implies that more acculturated individuals have higher susceptibility to health risks compared with their less acculturated counterparts^39,40^. For example, recent Chinese immigrants report a healthier diet and more physical activity than those who have resided in the United States >10 years^42^. Likewise, recent Japanese immigrants to the United States often adhere to a traditional Japanese lifestyle, including, but not limited to, healthful dietary practices and physical activity, and often have lower CVD rates and risk factors compared to those of Japanese ethnicity who were born outside of Japan^43^.

Our finding that second-generation Indians have an increased risk of incident CVD and its risk traits, particularly hyperlipidemia, independent of conventional CVD risk factors, suggest that being born in a developed country like Singapore with a “high-risk” lifestyle such as sedentary employment, consumption of more energy-dense processed foods, lack of physical activity, and psychological stress, may be the contributing factors^44,45^. These exposures may make them more susceptible to an increased risk of developing CVD and its risk attributes. However, as we did not collect these and other variables such as psychological stress, depression, dietary practices and physical activity in SINDI, further longitudinal studies are needed to confirm this hypothesis. Nonetheless, our results indicate that public health actions to address these detrimental modifications in lifestyle in second-generation individuals and prevent an inordinate increase in CVD outcomes.

One important factor that may be contributing to our finding that second-generation Indian individuals had higher risk of incident CVD and its risk traits (hyperlipidemia and CKD) is that our second-generation individuals had lower SES. Numerous studies have shown poor SES predisposes individuals to a higher risk of depression^46^, high psychiatric morbidity^47^, more disability^48^ and poorer healthcare access^49^. Many risk factors for CVD are also associated with low SES^50^. Therefore, it is possible that socio-economic disadvantage played a role in the development of CVD and its risk factors in second-generation individuals in our cohort. However, as our fully adjusted model showed that generational impact on incidence of CVD and its risk factors was independent of SES, further work is needed to confirm this finding.

Strengths of our study include a population-based and prospective study design, the use of standardized interview-based assessment and clinical testing protocols, and inclusion of a wide range of CVD risk factors. However, our results must be interpreted within the context of certain limitations. The assessment of CVD was self-reported and, as a result, our outcome may be subjected to recall and other biases. However, we believe that that the impact of such biases would likely be similar in first and second-generation Indians. In addition, previous studies have shown self-reported outcomes for CVD to be valid in the estimation of actual outcome^51–53^. Combined with our reliability results of ~75%, we consider that the self-reported CVD outcomes captured in our study remains an appropriate indicator of actual CVD events. Another limitation is that our hypothesis assumes that first-generation Indian immigrants had different lifestyles or environments in their home country before moving to Singapore. This assumption may not hold true and, as such, caution should be taken when interpreting our results. Last, information on risk factors, such as psychological stress, dietary practices, physical inactivity, are not available in this study, and therefore the possibility of residual confounding in our regression analysis could not be excluded. Further studies to elucidate the role of these factors are warranted.

In summary, we showed that second-generation Indians have increased risk of incident CVD, hyperlipidemia and CKD, compared to first-generation immigrants, after controlling for a range of conventional CVD risk factors. This information can inform key policy- and decision-makers in design and implementation of targeted CVD and its risk factors preventative strategies in susceptible second-generation Indian individuals.

## Electronic supplementary material

Supplementary Table S1

## References

[1] Roth GA (2015). Global and regional patterns in cardiovascular mortality from 1990 to 2013. Circulation.

[2] Akil L, Ahmad HA (2012). Relationships between obesity and cardiovascular diseases in four southern states and Colorado. J health Care Poor Underserved.

[3] Kachur S, Lavie CJ, de Schutter A, Milani RV, Ventura HO (2017). Obesity and cardiovascular diseases. Minerva Med.

[4] Prasad DS, Das BC (2009). Physical inactivity: a cardiovascular risk factor. Indian J Med Sci.

[5] Riha J (2014). Urbanicity and lifestyle risk factors for cardiometabolic diseases in rural Uganda: a cross-sectional study. PLoS Med.

[6] Hernandez AV, Pasupuleti V, Deshpande A, Bernabe-Ortiz A, Miranda JJ (2012). Effect of rural-to-urban within-country migration on cardiovascular risk factors in low- and middle-income countries: a systematic review. Heart (British Cardiac Society).

[7] Steyn, K. & Damasceno, A. Lifestyle and Related Risk Factors for Chronic Diseases. In: Jamison, D. T. *et al*., editors. Disease and Mortality in Sub-Saharan Africa. Washington (DC): World Bank The International Bank for Reconstruction and Development/The World Bank.; 2006. 21290651

[8] Marmot MG, Syme SL (1976). Acculturation and coronary heart disease in Japanese-Americans. Am J Epidemiol.

[9] Lindstrom M, Sundquist J (2001). Immigration and leisure-time physical inactivity: a population-based study. Ethn Health.

[10] Delavari M, Sonderlund AL, Swinburn B, Mellor D, Renzaho A (2013). Acculturation and obesity among migrant populations in high income countries–a systematic review. BMC Public Health.

[11] Holmboe-Ottesen Gerd, Wandel Margareta (2012). Changes in dietary habits after migration and consequences for health: a focus on South Asians in Europe. Food & Nutrition Research.

[12] Lesser IA, Gasevic D, Lear SA (2014). The association between acculturation and dietary patterns of South Asian immigrants. PloS One.

[13] Gadd M, Sundquist J, Johansson SE, Wandell P (2005). Do immigrants have an increased prevalence of unhealthy behaviours and risk factors for coronary heart disease?. Eur J Cardiovasc Prev Rehabil.

[14] Zheng Y (2012). Impact of migration and acculturation on prevalence of type 2 diabetes and related eye complications in Indians living in a newly urbanised society. PloS One.

[15] Zheng Y (2012). Prevalence and risk factors of diabetic retinopathy in migrant Indians in an urbanized society in Asia: the Singapore Indian eye study. Ophthalmology.

[16] Ueshima H (2003). Differences in cardiovascular disease risk factors between Japanese in Japan and Japanese-Americans in Hawaii: the INTERLIPID study. J Hum Hypertens.

[17] Sundquist J, Winkleby M (2000). Country of birth, acculturation status and abdominal obesity in a national sample of Mexican-American women and men. Int J Epidemiol.

[18] Sundquist J, Winkleby MA (1999). Cardiovascular risk factors in Mexican American adults: a transcultural analysis of NHANES III, 1988-1994. Am J Public Health.

[19] Lutsey PL (2008). Associations of acculturation and socioeconomic status with subclinical cardiovascular disease in the multi-ethnic study of atherosclerosis. Am J Public Health.

[20] Morales LS, Leng M, Escarce JJ (2011). Risk of cardiovascular disease in first and second generation Mexican-Americans. J Immigr Minor Health.

[21] Singh GK, Siahpush M (2001). All-cause and cause-specific mortality of immigrants and native born in the United States. Am J Public Health.

[22] Lopez L (2014). Impact of acculturation on cardiovascular risk factors among elderly Mexican Americans. Ann Epidemiol.

[23] Jin K, Gullick J, Neubeck L, Koo F, Ding D (2017). Acculturation is associated with higher prevalence of cardiovascular disease risk-factors among Chinese immigrants in Australia: Evidence from a large population-based cohort. Eur J Prev Cardiol.

[24] Chiu M, Maclagan LC, Tu JV, Shah BR (2015). Temporal trends in cardiovascular disease risk factors among white, South Asian, Chinese and black groups in Ontario, Canada, 2001 to 2012: a population-based study. BMJ Open.

[25] Kurian AK, Cardarelli KM (2007). Racial and ethnic differences in cardiovascular disease risk factors: a systematic review. Ethn Dis.

[26] Inkpen, C. 7 facts about world migration. Pew Research Center. Available at: http://www.pewresearch.or/fact-tank/2014/09/02/7-facts-about-world-migration/ (2014).

[27] The Singapore Department of Statistics. Singapore Population and Population Structure. Available at: http://www.singstat.gov.sg/statistics/browse-by-theme/population-and-population-structure.

[28] Singapore Department of Statistics. Singapore in figures 2010. Ethnic distribution, 2009. Available at: http://www.simgstat.gov.sg/pubn/popn/c2010acr/key_demographic_trends.pdf.

[29] Lavanya R (2009). Methodology of the Singapore Indian Chinese Cohort (SICC) eye study: quantifying ethnic variations in the epidemiology of eye diseases in Asians. Ophthalmic Epidemiol.

[30] Sabanayagam C (2017). Singapore Indian Eye Study-2: methodology and impact of migration on systemic and eye outcomes. Clin Exp Ophthalmol.

[31] Wong MYZ (2017). Is Corneal Arcus Independently Associated With Incident Cardiovascular Disease in Asians?. Am J Ophthalmol.

[32] Standards of medical care in diabetes–2015: summary of revisions. *Diabetes care* **38** Suppl, S4 (2015). 10.2337/dc15-S003 25537706

[33] National High Blood Pressure Education P. The Seventh Report of the Joint National Committee on Prevention, Detection, Evaluation, and Treatment of High Blood Pressure. Bethesda (MD): National Heart, Lung, and Blood Institute (US); 2004. 20821851

[34] Levey AS (2009). A new equation to estimate glomerular filtration rate. Ann Intern Med.

[35] Lim CC (2015). Chronic kidney disease, cardiovascular disease and mortality: A prospective cohort study in a multi-ethnic Asian population. Eur J Prev Cardiol.

[36] Le-Scherban F (2016). Immigrant status and cardiovascular risk over time: results from the Multi-Ethnic Study of Atherosclerosis. Ann Epidemiol.

[37] Moon JR (2012). Stroke incidence in older US Hispanics: is foreign birth protective?. Stroke.

[38] Abraido-Lanza AF, Dohrenwend BP, Ng-Mak DS, Turner JB (1999). The Latino mortality paradox: a test of the “salmon bias” and healthy migrant hypotheses. Am J Public Health.

[39] Dixon LB, Sundquist J, Winkleby M (2000). Differences in energy, nutrient, and food intakes in a US sample of Mexican-American women and men: findings from the Third National Health and Nutrition Examination Survey, 1988-1994. Am J Epidemiol.

[40] Diez Roux AV (2005). Acculturation and socioeconomic position as predictors of coronary calcification in a multiethnic sample. Circulation.

[41] Fang CY, Ross EA, Pathak HB, Godwin AK, Tseng M (2014). Acculturative stress and inflammation among Chinese immigrant women. Psychosom Med.

[42] Taylor VM (2007). Heart disease prevention among Chinese immigrants. J Community Health.

[43] Robertson TL (1977). Epidemiologic studies of coronary heart disease and stroke in Japanese men living in Japan, Hawaii and California. Coronary heart disease risk factors in Japan and Hawaii. Am J Cardiol.

[44] Arandia G, Nalty C, Sharkey JR, Dean WR (2012). Diet and acculturation among Hispanic/Latino older adults in the United States: a review of literature and recommendations. J Nutr Gerontol Geriatr.

[45] Murillo R, Albrecht SS, Daviglus ML, Kershaw KN (2015). The Role of Physical Activity and Sedentary Behaviors in Explaining the Association Between Acculturation and Obesity Among Mexican-American Adults. Am J Health Promot.

[46] Freeman A (2016). The role of socio-economic status in depression: results from the COURAGE (aging survey in Europe). BMC public health.

[47] Lazzarino AI, Hamer M, Stamatakis E, Steptoe A (2013). Low socioeconomic status and psychological distress as synergistic predictors of mortality from stroke and coronary heart disease. Psychosom Med.

[48] Wamala SP (2001). Large social inequalities behind women’s risk of coronary disease. Unskilled work and family strains are crucial factors. Lakartidningen..

[49] Kristiansson C (2009). Access to health care in relation to socioeconomic status in the Amazonian area of Peru. Int J Equity Health.

[50] Winkleby MA, Jatulis DE, Frank E, Fortmann SP (1992). Socioeconomic status and health: how education, income, and occupation contribute to risk factors for cardiovascular disease. Am J Public Health.

[51] Eliassen BM (2016). Validity of self-reported myocardial infarction and stroke in regions with Sami and Norwegian populations: the SAMINOR 1 Survey and the CVDNOR project. BMJ open.

[52] Wada K (2009). Self-reported medical history was generally accurate among Japanese workplace population. J Clin Epidemiol.

[53] Yamagishi K, Ikeda A, Iso H, Inoue M, Tsugane S (2009). Self-reported stroke and myocardial infarction had adequate sensitivity in a population-based prospective study JPHC (Japan Public Health Center)-based Prospective Study. J Clin Epidemiol.

