# Supplementary material for: Risk of Incident Cardiovascular Disease and Cardiovascular Risk Factors in First and Second-Generation Indians: The Singapore Indian Eye Study
Source: Sci Rep. 2018 Oct 4;8:14805. doi: 10.1038/s41598-018-32833-0 (PMC6172283; doi:10.1038/s41598-018-32833-0)
Supplement: Supplementary file 1 — Supplementary Table S1 [file 41598_2018_32833_MOESM1_ESM.docx]

**Risk of Incident Cardiovascular Disease and Cardiovascular Risk Factors in First and Second-Generation Indians: The Singapore Indian Eye Study**

**Running Head:** Generational influence on Incident CVD and risk factors

Preeti Gupta, PhD^1^, Alfred Tau Liang Gan, MSc^1^, Ryan Eyn Kidd Man, PhD^1^, Eva K. Fenwick, PhD^1,2^, Yih-Chung Tham, PhD^1^, Charumathi Sabanayagam, MD, PhD^1,2^, Tien Yin Wong, MD, PhD^1,2,3,4^, Ching-Yu Cheng MD, PhD^1,2,3,4^, Ecosse L. Lamoureux*, PhD^1,2,4^

1. Singapore Eye Research Institute, Singapore
2. Duke-NUS Medical School, Singapore
3. Singapore National Eye Centre, Singapore
4. National University of Singapore, Dept. of Ophthalmology, Singapore

**Corresponding author:**

Professor Ecosse L. Lamoureux,

Singapore Eye Research Institute (SERI)

DID: (+65) 6576 7382

**Email:** ecosse.lamoureux@seri.com.sg

| **Supplementary Table S1.** Association between years lived in Singapore and incidence of CVD and related conditions | | |
| --- | --- | --- |
| **Conditions** | **Relative risk (per 10 years lived)* (95% CI)** | **P value** |
| Myocardial infarction | 1.39 (1.00 to 1.93) | **0.050** |
| Stroke | 1.00 (0.72 to 1.38) | 0.979 |
| Angina | 1.12 (0.74 to 1.68) | 0.591 |
| CVD | 1.25 (1.00 to 1.57) | 0.054 |
| Diabetes | 0.99 (0.88 to 1.11) | 0.828 |
| Hypertension | 1.06 (0.98 to 1.15) | 0.152 |
| Hyperlipidaemia | 1.12 (1.05 to 1.20) | **0.001** |
| CKD | 1.26 (1.07 to 1.48) | **0.005** |
| Obesity | 1.04 (0.89 to 1.22) | 0.589 |
| *Adjusted for age, gender, obesity, low SES (primary or lower education, and individual monthly income < SGD2000), smoking, alcohol use, anti-diabetic, anti-hypertensive, and anti-cholesterol medications use, and mutually for the other CVD related conditions at baseline  Bolded values indicate statistically significant results  CVD = cardio vascular disease; CKD = chronic kidney disease; SES = socioeconomic status | | |
